# Supplementary material for: Bis-allylic Deuterated DHA Alleviates Oxidative Stress in Retinal Epithelial Cells
Source: Antioxidants (Basel). 2019 Oct 1;8(10):447. doi: 10.3390/antiox8100447 (PMC6826779; doi:10.3390/antiox8100447)
Supplement: Supplementary file 1 [file antioxidants-08-00447-s001.pdf]

Supporting Information

Bis-allylic deuterated DHA alleviates oxidative stress in retinal epithelial cells

Mélissa Rosell[1], Martin Giera[2], Philippe Brabet[3], Mikhail S. Shchepinov[4], Michel Guichardant[5], Thierry Durand[1], Joseph Vercauteren[1], Jean-Marie Galano[1] and Céline Crauste[1]

[1] IBMM, Univ Montpellier, CNRS, ENSCM, Montpellier, France, celine.crauste@umontpellier.fr

[2] Leiden University Medical Center, Center for Proteomics and Metabolomics, Albinusdreef 2, 2333ZA Leiden, The Netherlands

[3] Institute for Neurosciences of Montpellier, INSERM U1051-UM, Hospital St Eloi, 80 rue Augustin Fliche, 34091 Montpellier, France

[4] Retrotope, Inc. Los Altos, CA 94022, USA

[5] Univ-Lyon, Inserm UMR 1060, Inra UMR 1397 (CarMeN Laboratory), IMBL, INSA-Lyon, Villeurbanne, France

I – Chemical synthesis of deuterated lipophenols
II – Choice of oxidative stress conditions of ARPE-19 cells
III- Validation of FACS analysis conditions
IV – Sample treatment of oxidase DHAs: extraction yield
V – MS/MS analysis of NeuroP(s)
VI - Influence of deuterium incorporation on 15s-LOX kinetics

I – Chemical synthesis of deuterated lipophenols

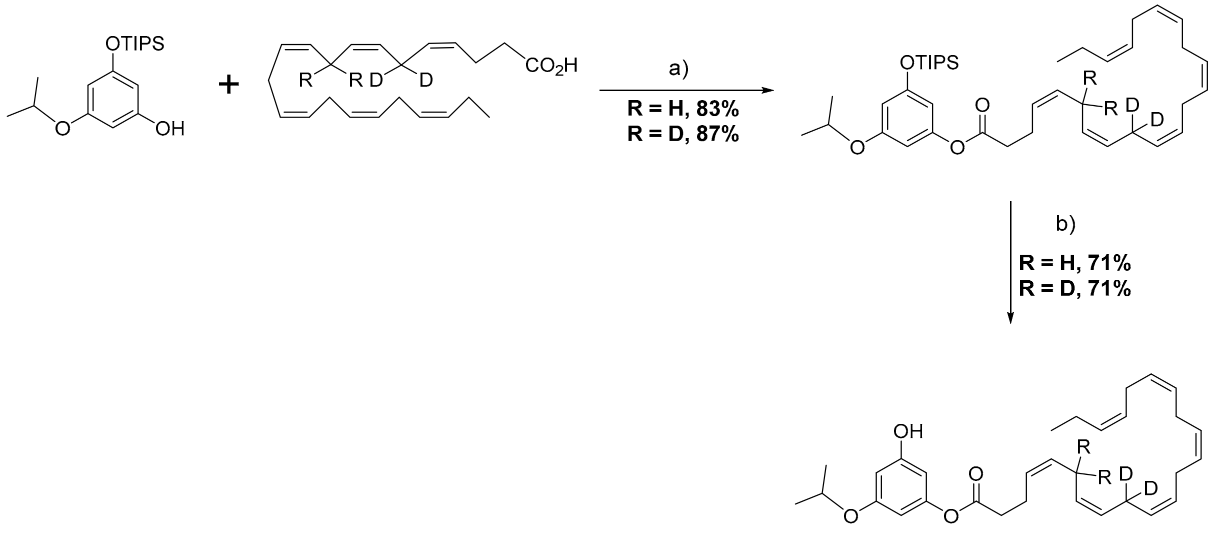

deuterated IP-DHA

Scheme S1. Synthesis of deuterated lipophenols: IP-D_2-DHA and IP-D_4-DHA.
a) DCC, DMAP, DCM. b) Et_3N-3HF, THF.

The deuterated DHA were previously synthesized according to the procedure described[1] and the synthesis of IP-D_2-DHA and IP-D_4-DHA was performed by using the procedure developed by Crauste et al.[2]

General procedure for the synthesis of deuterated IP-DHA protected by TIPS
5-Isopropoxy-1,3-bis(triisopropylsilyloxy)benzene[2] (75 mg, 0.23 mmol) dissolved in dry CH_2Cl_2 (3.7 mL) was added to a solution of deuterated DHA (0.21 mmol) in dry CH_2Cl_2 (3.7 mL) at room temperature under inert atmosphere. Then DCC (47 mg, 0.23 mmol) and DMAP (2 mg, 0.02 mmol) were added to the mixture. The reaction was stirred for 2 h and then additional DCC (22 mg, 0.1 mmol) and DMAP (2 mg, 0.02 mmol) were added. After 4 h the reaction was cooled at 4°C for 1 h to induce the crystallization of dicyclohexylurea. The mixture was then filtered to remove the solid. The filtrate was recovered and the solvent was removed under reduce pressure. The residue was purified by chromatography on silica gel (pentane/EtOAc, 99:1) to give the coupled product as a pale yellow oil.

IP-D_2-DHA protected by TIPS: (111 mg, 83% yield)
R_f=0.55 (pentane/EtOAc, 95:5);
^{1}H NMR (500 MHz, $CDCl_3$): δ = 6.28–6.27 (m, 1H, CH_{Ar}), 6.23–6.22 (m, 1H, CH_{Ar}), 6.21–6.20 (m, 1H, CH_{Ar}), 5.47–5.28 (m, 12H, $CH_{vinylic}$), 4.44 (septuplet, ^3J(H,H)=6Hz, 1H, CH_{OiPr}), 2.86–2.80 (m, 8H, $CH_{2(bis\text{-}allylic)}$, 2.60–2.57 (m, 2H, CH_2–CO), 2.51–2.48 (m, 2H, $\underline{CH_2}$– CH_2–CO), 2.10–2.04 (m, 2H,$\underline{CH_2}$-CH_3), 1.30 (d, ^3J(H,H)=6Hz, 6H, $CH_{3(OiPr)}$), 1.27–1.21 (m, 3H,CH_{TIPS}), 1.09 (d, ^3J(H,H)=7.5Hz, 18H, $CH_{3(TIPS)}$), 0.97 (t, ^3J(H,H)=8.5Hz, 3H, CH_3) ppm;
^{13}C NMR (125 MHz, $CDCl_3$): δ = 171.4, 159.3, 157.5, 152.1, 132.1, 129.6, 128.7, 128.5, 128.4, 128.4, 128.2, 128.2, 128.0, 128.0, 127.8, 127.1, 106.1, 105.5, 102.4, 70.3, 34.4, 25.8, 25.8, 25.7, 25.7, 25.1 (quint., ^1J(C,D)=19.2 Hz), 22.9, 22.1 (2C), 20.7, 18.0 (6C), 14.4, 12.7 (3C) ppm;
HRMS (ASAP$^+$-TOF): *m/z*: calcd for $C_{40}H_{61}D_2O_4Si$: 637.4621 [M + H]$^+$; found: 637.4617.

IP-D_4-DHA protected by TIPS: (84 mg, 87% yield)
R_f=0.56 (pentane/EtOAc, 95:5);
^{1}H NMR (500 MHz, $CDCl_3$): δ = 6.29–6.28 (m, 1H, CH_{Ar}), 6.23–6.22 (m, 1H, CH_{Ar}), 6.21–6.20 (m, 1H, CH_{Ar}), 5.47–5.28 (m, 12H, $CH_{vinylic}$), 4.44 (septuplet, ^3J(H,H)=6Hz, 1H, CH_{OiPr}), 2.86–2.80 (m, 6H, $CH_{2(bis\text{-}allylic)}$, 2.60–2.57 (m, 2H, CH_2–CO), 2.52–2.48 (m, 2H, $\underline{CH_2}$– CH_2–CO), 2.10–2.05 (m, 2H,$\underline{CH_2}$-CH_3), 1.30 (d, ^3J(H,H)=6Hz, 6H, $CH_{3(OiPr)}$), 1.27–1.21 (m, 3H,CH_{TIPS}), 1.09 (d, ^3J(H,H)=7.5Hz, 18H, $CH_{3(TIPS)}$), 0.97 (t, ^3J(H,H)=8.5Hz, 3H, CH_3) ppm;
^{13}C NMR (125 MHz, $CDCl_3$): δ = 171.3, 159.2, 157.5, 152.1, 132.1, 129.6, 128.7, 128.4, 128.4 (2C), 128.2, 128.1, 128.1, 128.0, 127.8, 127.1, 106.1, 105.5, 102.4, 70.2, 34.4, 25.8, 25.7, 25.7, 25.1 (quint., ^1J(C,D)=19.4 Hz, 2C), 22.9, 22.1 (2C), 20.7, 18.0 (6C), 14.4, 12.7 (3C) ppm;
HRMS (ASAP$^+$-TOF): *m/z*: calcd for $C_{40}H_{59}D_4O_4Si$: 639.4747 [M + H]$^+$; found: 639.4742.

General procedure for the synthesis of deuterated IP-DHA

$Et_3N.3HF$ (83 µL, 0.51 mmol) was added dropwise to a solution of the deuterated DHA protected by TIPS (0.17 mmol) in dry THF (7.0 mL) at room temperature under inert atmosphere. Additional $Et_3N.3HF$ (55 µL, 0.34 mmol) was added after 2.5 h and 5.5 h of reaction. After 7 h, the reaction was quenched by adding EtOAc (20 mL) and H_2O (20 mL). The layers were separated and the aqueous one was extracted with 2×10 mL of EtOAc. The combined organic layers were dried over Na_2SO_4, filtered and the solvents removed under reduce pressure. The residue was purified by chromatography on silica gel (pentane/EtOAc, 95:5 to 85:15) to give the residue which was then purified by preparative HPLC (column Waters SymmetryPrep™ (C_{18}) 7 µm (7.8×300mm); H_2O/acetonitrile, 1:9 isocratic; detection 214 nm) to give the deuterated IP-DHA as a pale yellow oil.

IP-D$_2$-DHA: (60 mg, 71% yield)
***R_f*=**0.25(pentane/EtOAc, 85:15);
^{1}H NMR (500 MHz, $CDCl_3$): *δ* = 6.23–6.22 (m, 1H, CH_{Ar}), 6.20–6.17 (m, 1H, CH_{Ar}), 6.14–6.13 (m, 1H, CH_{Ar}), 5.47–5.30 (m, 12H, $CH_{vinylic}$), 4.44 (septuplet, ^3J(H,H)=6Hz, 1H, CH_{OiPr}), 2.85–2.80 (m, 8H, $CH_{2(bis\text{-allylic})}$, 2.60–2.58 (m, 2H, CH_2–CO), 2.52–2.48 (m, 2H, $\underline{CH_2}$– CH_2–CO), 2.10–2.04 (m, 2H,$\underline{CH_2}$-CH_3), 1.30 (d, ^3J(H,H)=6Hz, 6H, $CH_{3(OiPr)}$), 0.97 (t, ^3J(H,H) = 7.5Hz, 3H, CH_3) ppm;
^{13}C NMR (125 MHz, $CDCl_3$): *δ* = 172.1, 159.7, 157.4, 152.2, 132.3, 129.9, 128.8, 128.6, 128.5, 128.5, 128.3, 128.3, 128.1 (2C), 127.7, 127.2, 101.9, 101.8, 101.2, 70.5, 34.5, 25.9, 25.8, 25.8, 25.7, 25.3 (quint., ^1J(C,D)=19.1Hz), 24.9, 23.0, 22.2, 20.8, 14.5 ppm;
HRMS (ASAP$^+$-TOF): *m/z*: calcd for $C_{31}H_{41}D_2O_4$: 481.3287 $[M + H]^+$; found : 481.3293;
HPLC column Bridge® BEH (C_{18}) 2.5 µm (2.1 x 100 mm); H_2O/MeOH, 20:80 isocratic; PDA detection (200-800 nm): t_R = 13.61 min.

IP-D$_4$-DHA: (45 mg, 71% yield)
R_f=0.23 (pentane/EtOAc, 85:15);
^{1}H NMR (500 MHz, $CDCl_3$): *δ* = 6.23–6.22 (m, 1H, CH_{Ar}), 6.18–6.17 (m, 1H, CH_{Ar}), 6.14–6.13 (m, 1H, CH_{Ar}), 5.47–5.30 (m, 12H, $CH_{vinylic}$), 4.44 (septuplet, ^3J(H,H)=6Hz, 1H, CH_{OiPr}), 2.88–2.80 (m, 6H, $CH_{2(bis\text{-allylic})}$, 2.62–2.58 (m, 2H, CH_2–CO), 2.52–2.48 (m, 2H, $\underline{CH_2}$– CH_2–CO), 2.10–2.04 (m, 2H,$\underline{CH_2}$-CH_3), 1.30 (d, ^3J(H,H)=6Hz, 6H, $CH_{3(OiPr)}$), 0.97 (t, ^3J(H,H) = 7.5Hz, 3H, CH_3) ppm;
^{13}C NMR (125 MHz, $CDCl_3$): *δ* = 171.9, 159.7, 157.3, 152.2, 132.2, 129.8, 128.7, 128.4 (3C), 128.2, 128.1, 128.0, 128.0, 127.6, 127.1, 101.8, 101.7, 101.1, 70.4, 34.4, 25.8, 25.7, 25.7, 25.1 (quint., ^1J(C,D)=19.4 Hz), 22.9, 22.1, 20.7, 14.4 ppm;
HRMS (ASAP$^+$-TOF): calcd. for $C_{31}H_{39}D_4O_4$: 483.3412 $[M + H]^+$; found: 483.3410;
HPLC column Bridge® BEH (C_{18}) 2.5 µm (2.1 x 100 mm); H_2O/MeOH, 20:80 isocratic; PDA detection (200-800 nm): t_R = 12.90 min.

(1) Rosell, M.; Villa, M.; Durand, T.; Galano, J.-M.; Vercauteren, J.; Crauste, C. Total Syntheses of Two Bis-Allylic-Deuterated DHA Analogues. *Asian J. Org. Chem.* **2017**, *6* (3), 322–334. https://doi.org/10.1002/ajoc.201600565.

(2) Crauste, C.; Vigor, C.; Brabet, P.; Picq, M.; Lagarde, M.; Hamel, C.; Durand, T.; Vercauteren, J. Synthesis and Evaluation of Polyunsaturated Fatty Acid–Phenol Conjugates as Anti-Carbonyl-Stress Lipophenols. *European Journal of Organic Chemistry* **2014**, *2014* (21), 4548–4561. https://doi.org/10.1002/ejoc.201402282.

II – Choice of oxidative stress conditions of ARPE-19 cells :

- ***Serum starvation at 1% FBSM*** *was used to provide sufficient and intermediary oxidative stress based on general oxidative status (DCFDA probe) and free radical processes involved in lipid peroxidation (C11-Bodipy$^{581/591}$ probe), compared to 2.5 % and 10% FBSM or serum free medium as described in* ***Fig. S1 and S2****.*
- First, the influence of serum in cell medium was evaluated and was found to strongly influence DHA toxicity on cells. DHAs showed toxicity on ARPE-19 cells only in serum-free medium or when using 1% FBSM (fetal bovine serum media), compared to 2.5% or 10% FBSM, where no DHA toxicity was observed up to a final concentration of 110 µM. FBS mediated the protective effect against pro-oxidant conditions, and was responsible for a decrease in global oxidative status (**Fig. S1,** DCFDA probe[3]) and in lipid peroxidation status (**Fig. S2**).

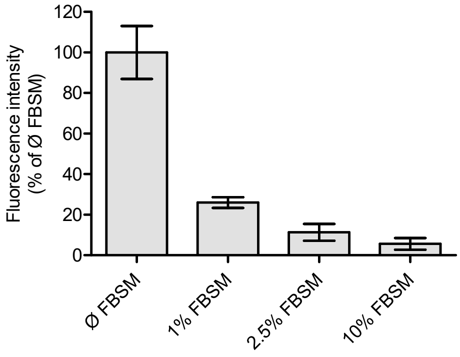

Figure S1. Intensity of fluorescence observed during DCFDA oxidation by radical species **on ARPE-19 cells in different medium (FACS analysis).** Radical reactive species able to oxidize DCFDA were measured in ARPE-19 cells. The cell permeant reagent DCFDA is deacetylated by cellular esterases to dichlorofluorescein (DCFH), which can be oxidized by several radical reactive species (peroxyl, alkoxyl, $NO_2^{\bullet}$, carbonate, $HO^{\bullet}$, …)[3] into the fluorophore 2′,7′–dichlorofluorescein (DCF). ARPE-19 cells were plated into 2 cm^2 wells (2×10^5 cells/well) and cultured for 24 h to reach confluence before drug treatment. The cell cultures were incubated with 2 µM of DCFDA for 40 min in serum free medium. The cells were rinsed and incubated in different media without phenol red (serum free, 1% FBSM, 2.5% FBSM or 10% FBSM) for 4 h followed by the FACS analysis. The data are expressed as the percentage of treated cells with serum free medium (Ø FBSM) and presented as means ± SD (n = 1 experiment, each condition in triplicate). 2′,7′–dichlorofluorescin diacetate (DCFDA) was purchased from Abcam and dissolved in DMSO to prepare stock solution at 20 mM.

(3) Halliwell, B.; Whiteman, M. Measuring Reactive Species and Oxidative Damage in Vivo and in Cell Culture: How Should You Do It and What Do the Results Mean? *Br J Pharmacol* **2004**, *142* (2), 231–255. https://doi.org/10.1038/sj.bjp.0705776.

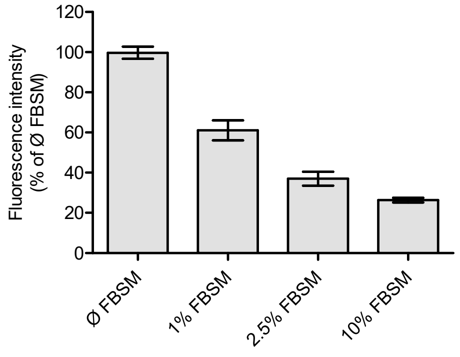

Figure S2. Intensity of C11-BODIPY fluorescence on ARPE-19 cells in different medium (FACS analysis). ARPE-19 cells were plated into 2 cm^2 wells (2×10^5 cells/well) and cultured for 24 h to reach confluence. The cells were always rinsed with the corresponding described medium before treatment. For all the experiments, DMEM/Ham F12 without phenol red was used. The cell cultures were incubated with 5 µM of C11-BODIPY$^{581/591}$ for 30 min in serum free medium. The cells were rinsed and incubated in the different media (serum free, 1% FBSM, 2.5% FBSM or 10% FBSM) for 24 h followed by the FACS analysis. The data are expressed as the percentage of treated cells with serum free medium (Ø FBSM) and presented as means ± SD (n = 1 experiment, each condition in triplicate).

- ***H_2O_2 concentration*** *used in toxicity assay (600 µM) lead to 60-70% of mortality (**Fig. S3**), whereas the one used during the comparison of lipid peroxidation status (400 µM) evaluate by FACS methodology, avoid important cell mortality and provide a plateau in global oxidative status (**Fig. S3 and S4**).*

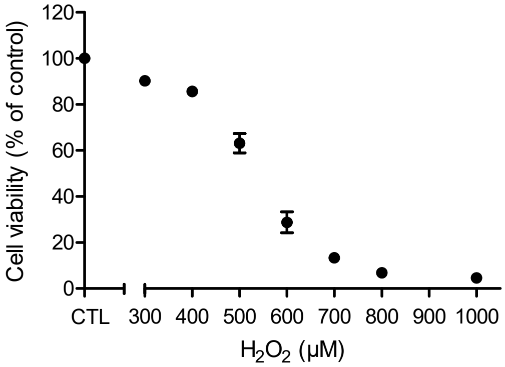

Figure S3. Toxicity of H_2O_2 in 1% FBSM on ARPE-19 cell. ARPE-19 cells were plated into 96-well plates (3×10^4 cells/well) and cultured for 24 h to reach confluence before H_2O_2 treatment. The cell cultures were treated in medium with 1% FBS (1% FBSM) containing H_2O_2 at different concentrations (0-1000 µm). The viability of the cells was determined in quadruplicate samples 4 h later, using a MTT colorimetric assay. Toxicity of H_2O_2 in 1% FBSM on ARPE-19 cells. The data are expressed as the percentage of control untreated cells and presented as means ± SEM (n = 4 independent experiments, each condition in quintuplicate).

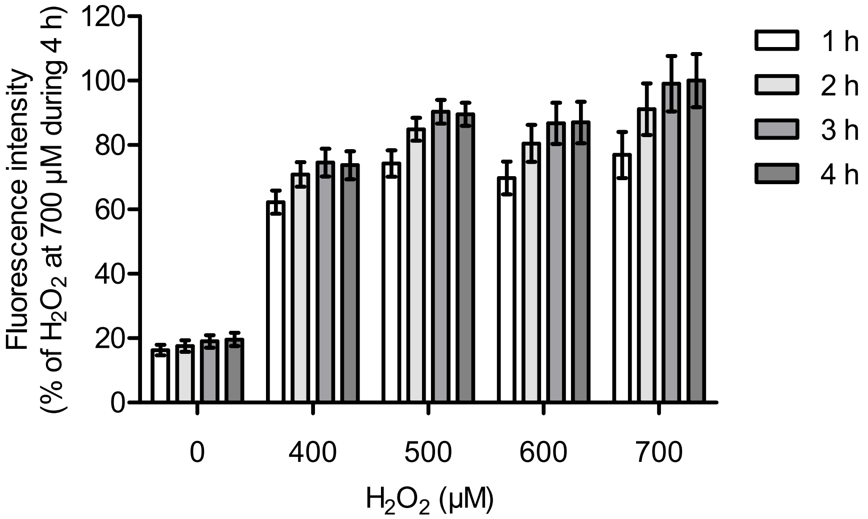

Figure S4. Intensity of fluorescence observed during DCFDA oxidation by reactive species assays on ARPE-19 cells in presence of different concentration of H_2O_2 in 1% FBSM (fluorometry analysis). ARPE-19 cells were put into black well, clear bottom 96-well View Plates (Perkin Elmer, USA) at 3×10^4cells/well and cultured for 24 h to reach confluence before oxidative stress. The medium was removed and the cells were washed with Dulbecco's Modified Eagle's Medium (DMEM)/Ham F12 without phenol red and incubated for 45 min at 37°C in the same medium containing 2 µM of DCFDA. The cells were then washed and treated with a 1% FBSM containing H_2O_2 at different concentrations (0–800 µm) for 4 h. DCF production was measured with a fluorescence spectrometer (CLARIO star, BMG LABTECH's) with excitation and emission wavelengths at 485 nm and 535 nm, respectively. The data are expressed as the percentage of treated cells with 700 µM of H_2O_2 during 4 h and presented as means ± SD (n = 1 experiment, each condition in quintuplicate). 2',7'–dichlorofluorescin diacetate (DCFDA) was purchased from Abcam and dissolved in DMSO to prepare stock solution at 20 mM.

III- Validation of FACS analysis conditions

- ***Light intensities*** *of 5 000 to 20 0000 lux for the photobleaching stress were also tested, and 5 000 lux was efficient to observed an increase of oxidized C11-Bodipy*$^{581/591}$ *(**Fig. S5**).*

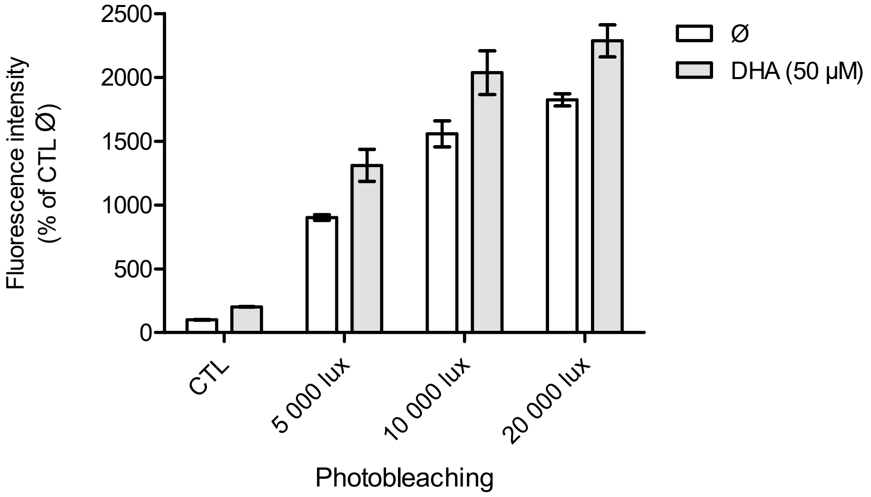

Figure S5. Intensity of C11-BODIPY Fluorescence on ARPE-19 cells with photobleaching at different intensities and DHA treatment (FACS analysis). ARPE-19 cells were plated into 2 cm^2 wells (2×10^5 cells/well) and cultured for 24 h to reach confluence. The cells were rinsed with 1% FBSM before treatment. For all the experiments, DMEM/Ham F12 without phenol red was used. The cell cultures were treated with 1% FBSM containing DHA at 50 µm for 24 h and then rinsed with 1% FBSM before the incubation of 5 µM C11-BODIPY$^{581/591}$ for 30 min. The cells were rinsed and exposed to a white LED lamp with different intensities (5 000, 10 000 or 20 000 lux) at room temperature during 1 h followed by the FACS analysis. The data are expressed as the percentage of untreated cells (CTL Ø) and presented as means ± SD (n = 1 experiment, each condition in triplicate).

- *The pre-treatment with natural DHA had no impact on the probe incorporation (**Fig. S6**).*

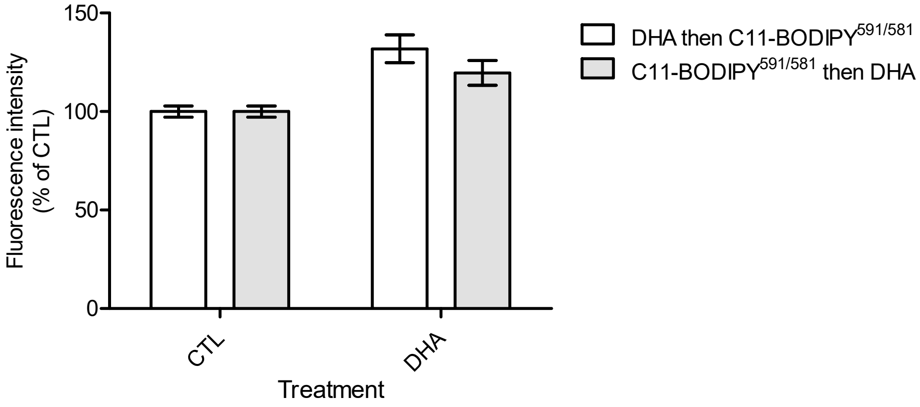

Figure S6. Intensity of C11-BODIPY Fluorescence on ARPE-19 cells: Influence of DHA (50 µM) on probe incorporation (FACS analysis). ARPE-19 cells were plated into 2 cm^2 wells (2×10^5 cells/well) and cultured for 24 h to reach confluence. The cells were always rinsed with 1% FBSM before treatment. For all the experiments DMEM/Ham F12 without phenol red was used. A part of the cells was treated with 1% FBSM containing DHA at 50 µm for 24 h, and then rinsed with media before the incubation of 5 µM C11-BODIPY$^{581/591}$ for 30 min. The other part of the cells was incubated with 1% FBSM containing 5 µM C11-BODIPY$^{581/591}$ for 30 min, then rinsed and treated with 1% FBSM containing DHA at 50 µm for 24 h. All the cells were finally rinsed and incubated in 1% FBSM during 24 h followed by the FACS analysis. The data are expressed as the percentage of untreated cells (CTL Ø) and presented as means ± SD (n = 1 experiment, each condition in triplicate).

IV – sample treatment of oxidase DHAs: extraction yield

$$\frac{Standard\ area\ prespiked}{Standard\ area\ postspiked} \times 100 = \ Extraction\ yield\ (\%)$$

		Extraction yield ± SD (%)
Internal standard	C21-15-F_{2t}-IsoP	83,23 ± 12,21
Neuroprostanes	4(*RS*)-F_{4t}-NeuroP	100,48 ± 22,93

Figure S7. Protocol and analysis of extraction yield. The preparation of the sample of oxidized natural DHA was validated through the evaluation of the yield of extraction for the SPE protocol. For this protocol, a monoplicat of the following solution was prepared: 1) Two samples of oxidized natural DHA (n = 2) were spiked with 4 ng of IS stock solution and with 3.2 ng of standards solution, and were extracted as previously described; 2) Two samples of oxidized natural DHA (n=2) were extracted then spiked with 4 ng of IS stock solution and with 3.2 ng of standards solution. All sets of two samples were analyzed once using the LC-MS/MS system. The yield of extraction was calculated as the percentage difference between peaks areas of standards in 1) pre-spiked and 2) post-spiked samples with each set of data.

V – MS/MS analysis of 4-F_{4t}-NeuroP(s)

Standards and Transitions	Q1 (m/z)	Q3 (m/z)	RT(min)	DP (V)	EP (V)	CE (V)	CXP (V)
C21_15F_{2t}-IsoP_T1	367.200	193.200	11.8	-174	-3.10	-34.00	-27.0
C21_15F_{2t}-IsoP_T2	367.200	261.300	11.8	-174	-3.10	-34.00	-27.0
4(*RS*)-F_{4t}-NeuroP_T1	377.200	101.000	11.9	-161	-4.69	-28.30	-6.9
4(*RS*)-F_{4t}-NeuroP_T2	377.200	271.300	11.9	-161	-4.69	-27.00	-16.0

Table S1. MRM transitions, retention times (RT) and voltages (DP: declustering potential, EP: entrance potential, CE: collision energy, CXP: collision cell exit potential) used for the detection and determination of standards (IS) IsoP (C21-15-F_{2t}-IsoP) and NeuroPs (4(*RS*)-F_{4t}-NeuroP).

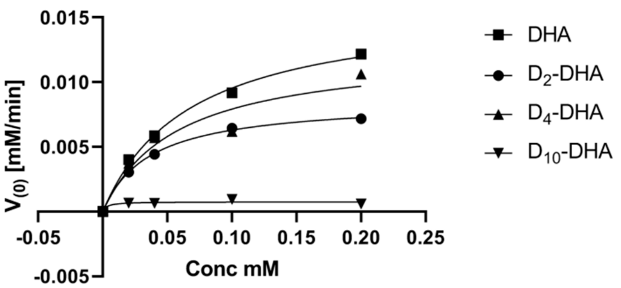

Figure S8. Influence of deuterium incorporation on 15s-LOX kinetics: Michaelis-Menten kinetics $V_{(0)}$ (initial reaction rates) derived from, UV analysis performed at 270 nm. The four investigated DHAs were tested at 0.02mM, 0.04mM, 0.1mM and 0.2mM, The 15-sLOX enzyme at 0.053 mg/mL. All $V_{(0)}$ values were determined in triplicate and the average was used for the determination of Michaelis-Menten kinetics of table 2.
